# Resistance Training with Co-ingestion of Anti-inflammatory Drugs Attenuates Mitochondrial Function

**DOI:** 10.3389/fphys.2017.01074

**Published:** 2017-12-19

**Authors:** Daniele A. Cardinale, Mats Lilja, Mirko Mandić, Thomas Gustafsson, Filip J. Larsen, Tommy R. Lundberg

**Affiliations:** ^1^Åstrand Laboratory, The Swedish School of Sport and Health Sciences, Stockholm, Sweden; ^2^Elite Performance Centre, Bosön—Swedish Sports Confederation, Lidingö, Sweden; ^3^Division of Clinical Physiology, Department of Laboratory Medicine, Karolinska Institutet, and Unit of Clinical Physiology, Karolinska University Hospital, Stockholm, Sweden

**Keywords:** acetylsalicylic acid, flywheel training, ibuprofen, OXPHOS, NSAID, strength training

## Abstract

**Aim:** The current study aimed to examine the effects of resistance exercise with concomitant consumption of high vs. low daily doses of non-steroidal anti-inflammatory drugs (NSAIDs) on mitochondrial oxidative phosphorylation in skeletal muscle. As a secondary aim, we compared the effects of eccentric overload with conventional training.

**Methods:** Twenty participants were randomized to either a group taking high doses (3 × 400 mg/day) of ibuprofen (IBU; 27 ± 5 year; *n* = 11) or a group ingesting a low dose (1 × 75 mg/day) of acetylsalicylic acid (ASA; 26 ± 4 year; *n* = 9) during 8 weeks of supervised knee extensor resistance training. Each of the subject's legs were randomized to complete the training program using either a flywheel (FW) device emphasizing eccentric overload, or a traditional weight stack machine (WS). Maximal mitochondrial oxidative phosphorylation (CI+II_P_) from permeabilized skeletal muscle bundles was assessed using high-resolution respirometry. Citrate synthase (CS) activity was assessed using spectrophotometric techniques and mitochondrial protein content using western blotting.

**Results:** After training, CI+II_P_ decreased (*P* < 0.05) in both IBU (23%) and ASA (29%) with no difference across medical treatments. Although CI+II_P_ decreased in both legs, the decrease was greater (interaction *p* = 0.015) in WS (33%, *p* = 0.001) compared with FW (19%, *p* = 0.078). CS activity increased (*p* = 0.027) with resistance training, with no interactions with medical treatment or training modality. Protein expression of ULK1 increased with training in both groups (*p* < 0.001). The increase in quadriceps muscle volume was not correlated with changes in CI+II_P_ (*R* = 0.16).

**Conclusion:** These results suggest that 8 weeks of resistance training with co-ingestion of anti-inflammatory drugs reduces mitochondrial function but increases mitochondrial content. The observed changes were not affected by higher doses of NSAIDs consumption, suggesting that the resistance training intervention was the prime mediator of the decreased mitochondrial phosphorylation. Finally, we noted that flywheel resistance training, emphasizing eccentric overload, rescued some of the reduction in mitochondrial function seen with conventional resistance training.

## Introduction

Although it is widely recognized that both high-volume and high-intensity (Granata et al., 2015; Vincent et al., 2015) aerobic exercise training is a potent stimulus for improving mitochondrial content and function (Holloszy, 1975), less is known about the effects of resistance training. It has been established that single bouts of resistance exercise can stimulate mitochondrial protein synthesis at least in the untrained state (Wilkinson et al., 2008). Yet, early research reported reduced mitochondrial content following resistance training, and this effect was attributed to hypertrophy-induced dilution of the mitochondrial fraction (MacDougall et al., 1979; Lüthi et al., 1986). Although conflicting findings exist (Tang et al., 2006), studies generally have reported decreased (Tesch et al., 1987), or unaltered (Wang et al., 1993; Lundberg et al., 2013; Alvehus et al., 2014) levels of oxidative enzymes such as citrate synthase (CS) activity, a marker of mitochondrial content (Larsen et al., 2012), following chronic resistance training. More recently, some emerging evidence infer that mitochondrial respiratory function is improved (Pesta et al., 2011; Irving et al., 2015; Porter et al., 2015) or unaltered (Robinson et al., 1995) by resistance training, yet the exact ability of resistance exercise to drive mitochondrial adaptations is still unclear (Groennebaek and Vissing, 2017). When comparing eccentric vs. concentric contractions, animal models have shown that mitochondrial respiration is mainly improved after concentric work (Isner-Horobeti et al., 2014). This could be due to the known fact that concentric work is much more energy consuming than eccentric work (Hoppeler, 2016), thereby triggering adaptations in the ATP-producing mitochondria. It remains to be explored how coupled concentric-eccentric muscle actions, favoring eccentric overload, affects mitochondrial adaptations.

Nonsteroidal anti-inflammatory drugs (NSAIDs) are among the most commonly consumed drugs in the world with a higher prevalence of usage in athletes and recreationally active people than in the rest of the population (Berglund, 2001; Warner et al., 2002). NSAIDs interfere with prostaglandin synthesis produced by the cyclooxygenase (COX) enzymes, which regulate numerous physiological processes (Smyth et al., 2009) including muscle protein metabolism and regeneration (Trappe et al., 2001, 2002; Mackey et al., 2016). Although past research has shown that NSAIDs might interfere with the increase in satellite cell activity (Mikkelsen et al., 2009), translational signaling (Markworth et al., 2014), and protein synthesis (Trappe et al., 2002) in response to acute resistance exercise, longitudinal training studies show equivocal results probably due to differences in drug dosage, length of training, and target population (Krentz et al., 2008; Petersen et al., 2011; Trappe et al., 2011; Jankowski et al., 2013).

In response to this uncertainty, we recently carried out an 8-week training study showing that maximal over-the-counter doses of ibuprofen compromised muscle strength and hypertrophic responses to resistance exercise in young healthy individuals (Lilja et al., 2017). However, little is known about the effects of NSAIDs on skeletal muscle mitochondrial adaptations. Apart from their classical function of controlling ATP production, mitochondria are also centrally involved in cell signal transduction, survival and homeostasis (Hepple, 2016). Although different pharmacological approaches have been shown to alter mitochondrial adaptations in animal and human models (Sanchis-Gomar et al., 2014; Suliman and Piantadosi, 2014; Tronstad et al., 2014), there are scarce reports on the effects of NSAID consumption. Reduced hepatic mitochondria oxidative phosphorylation was shown in rats exposed to NSAIDs (Mahmud et al., 1996). Specifically, NSAIDs may inhibit β-oxidation (Browne et al., 1999) and acyl-CoA synthetases (Kasuya et al., 2013) in mitochondria from mouse brain and liver. Furthermore, NSAIDs have been reported to increase mitochondria-derived reactive oxygen species (ROS) and cause cell damage in humans (Handa et al., 2014). In support, *in vitro* animal cell lines studies indicate that NSAIDs might uncouple mitochondrial respiration, inhibit ATP synthesis, and cause mitochondrial liver toxicity (Krause et al., 2003; Syed et al., 2016). Acutely, in humans, NSAIDs interact with mitochondrial membrane phospholipids, resulting in inhibition of mitochondrial electron transport and ATP production, and attenuated mitochondrial respiration capacity primarily at complex I (Boushel et al., 2012). Thus, the collective evidence suggests that NSAIDs may act locally within the muscle fibers to impair skeletal muscle mitochondrial metabolism.

In the only study so far examining NSAID intake and metabolic adaptations to resistance exercise, it was found that, in the elderly, 12 weeks of resistance training with COX-inhibiting drugs lead to an increase in CS activity compared to resistance training with placebo (Trappe et al., 2016). As the drug group also gained more muscle mass than the placebo group, the authors attributed the increased CS activity to the substantially greater growth of the metabolically superior type I fibers. This report was the first evidence to support the hypothesis that prostaglandins may regulate protein turnover not only in the myofibrillar protein pool, but also in the mitochondrial fraction. However, given the negative effects of high doses of NSAIDs on muscle growth in young subjects (Lilja et al., 2017), we hypothesized that a high dose of NSAIDs, potent enough to reach the peripheral tissue, would interfere with mitochondrial adaptations in response to resistance training. In contrast, low doses of acetylsalicylic acid should exert its irreversible anti-platelet effect with no profound effect on the peripheral tissue (Rosenkranz and Frölich, 1985; Capone et al., 2004).

To this background, the aim of the current study was to assess the effects of high vs. low doses of NSAID intake during 8 weeks of resistance training on mitochondrial oxidative phosphorylation activating different electron transfer complexes (mitochondrial function), and CS activity (a proxy of mitochondrial content) as well as on mitochondrial proteins, in young healthy subjects. A secondary aim was to assess the effects of eccentric overload compared with conventional resistance training. The overall hypothesis was that high NSAID doses would decrease both mitochondrial content and function.

## Materials and methods

### General design

This study was a single-blind randomized controlled training study with parallel groups. Moderately active men and women (a subset of the sample from the study by Lilja et al., 2017) were pair-matched based on strength and then randomized to either a group receiving high doses (3 × 400 mg/day) of ibuprofen [IBU; *n* = 11 (7 men, 4 women); 27 ± 5 years, body mass 76.6 ± 11.4 kg, height 172.6 ± 8 cm] or a group getting a low dose (1 × 75 mg/day) of acetylsalicylic acid (ASA; *n* = 9 (6 men, 3 women); 26 ± 4 years, body mass 83.7 ± 25.6 kg, height 177.7 ± 13.7 cm) during 8 weeks in combination with resistance training of the knee extensor muscles. Maximal mitochondrial oxidative phosphorylation (CI+II_P_) from permeabilized skeletal muscle bundles was assessed in high-resolution respirometry before and after the training intervention. Citrate synthase activity was assessed using spectrophotometry. Total protein content of targeted proteins was quantified by immunoblot analysis. Muscle quadriceps femoris volume was assessed by magnetic resonance imaging (MRI).

All volunteers were determined healthy based on a health screening survey and analysis of blood biochemistry. Subjects who had performed structured resistance training (more than once per week) for the past 6 months prior to the start of this study were excluded. Volunteers were informed about the possible risks and discomfort involved in the experiments before giving their written informed consent to participate. The study was approved by the Regional Ethical Review Board in Stockholm, Sweden. Procedures were undertaken in agreement with the Declaration of Helsinki and the study was registered as a clinical trial on ClinicalTrials.gov with the identifier NCT02531451. Methodological details and related findings from the larger study have been presented previously (Lilja et al., 2017).

### Drug administration and accountability

The subjects received the assigned drugs together with a drug-diary on the first day of consumption. The IBU group was instructed to take 3 doses/day (~08:00, ~14:00, and ~20:00) corresponding to the maximal over-the-counter daily dose of 1,200 mg (400 mg/dose). The ASA group receiving acetylsalicylic acid was instructed to take one dose/day (75 mg) in conjunction with their morning meal. Subjects were reminded through regular text messages to take their assigned drug. The drug diaries together with the remaining drugs were collected at the end of the 8-week intervention for drug compliance assessment. All medicine administration and accountability was performed in agreement with the code of Good Clinical Practice (ICH-GCP) by a certified external partner, the Clinical Pharmacology Trial Unit, at the Karolinska University Hospital in Huddinge, Stockholm. Despite these careful procedures, it is obviously impossible to assure full drug compliance since personal supervision of drug intake was not practically possible to implement. Although the subjects were not blinded to the treatment, the research team stayed blinded for the drug assignment during the whole intervention.

We chose to give the control group a very low but active dose of ASA in order to factor out the potential role of COX-inhibition in circulating platelets, which would be achieved by both IBU and ASA. Because of substantial pre-systemic clearance of ASA, exposure of platelets to the concentrations of the drug in the portal circulation leads to greater effects on the platelets than on systemic targets. As a consequence, much higher doses of ASA are needed to inhibit COX in the systemic vascular and peripheral tissue than in platelets, especially when administered orally. This is supported by research showing that low doses of ASA results in a cumulative inhibition of platelet TXA2, leaving vascular biosynthesis of prostacyclin, predominantly by systemic endothelium, intact (Weksler et al., 1983). Furthermore, the administration of low-dose ASA did not affect PGE_2_ production or prostacyclin biosynthesis in whole blood (Capone et al., 2004), and, as no significant reduction in the urinary 6-keto-PGF production was observed at doses of 50 and 100 mg of aspirin, it appeared that these doses did not inhibit systemic COX levels (Vial et al., 1990). In fact, plasma aspirin concentrations obtained after doses of 100 mg and less may sometimes be below the detection limit of the analytical method (Vial et al., 1990). In a comparison between 1,500 and 100 mg aspirin, it was concluded that low-dose ASA can effectively inhibit platelet function without producing pharmacologically active concentrations in the peripheral circulation (Rosenkranz and Frölich, 1985). The dose in the current study (75 mg) is also more than 10 times lower than used in the recent study by Ratchford et al. where muscle prostaglandin levels were unchanged or only modestly suppressed by 975 mg aspirin (Ratchford et al., 2017).

### Exercise equipment and training intervention

The training protocols and equipment have been described in detail elsewhere (Lilja et al., 2017). Briefly, each of the subject's legs were randomized (counterbalanced for dominance) either to a traditional weight stack device (WS) (World Class, Stockholm, Sweden) or a flywheel training device (FW) (YoYo Technology Inc., Stockholm, Sweden). The use of FW allows for maximal voluntary force to be produced from the first repetition, whereas WS is dependent on constant loading and hence maximal voluntary force is only offered in the last repetitions (i.e., close to failure). Further, the use of FW results in brief episodes of eccentric overload where the eccentric force exceeds peak concentric force (Tesch et al., 2004). The 8-week training intervention, targeting the knee extensor muscles, consisted of 20 training sessions scheduled 2 or 3 times every week. A standardized warm-up of 2 × 5 repetitions per leg in the FW and 1 × 5 in the WS was performed at the beginning of each session. After a 2 min rest, the subjects performed 4 × 7 maximal repetitions in the FW (2 min rest) using the leg that had been randomized as “FW leg” and 4 × 8–12 repetitions to failure in the WS, with the leg randomized as “WS leg.” The starting order of the machines was altered in between every training session.

### Magnetic resonance imaging

Cross-sectional images were obtained using a 1.5-Tesla Siemens Magnetom Aera (Siemens Healthcare, Germany) unit as previously described (Lilja et al., 2017). Subjects were resting in the supine position for 1 h prior to scan to avoid any influence of fluid shifts on muscle volume. Fifty continuous images with 10-mm slice thickness were obtained for each subject before and 6 days after the training intervention ended. CSA of the whole quadriceps femoris muscle was analyzed from the first image not displaying muscle gluteus maximus and ending with the last image in which muscle rectus femoris appeared. Within this segment (range 9–18 images), every third image was assessed by manual planimetry using imaging software (Image J, National Institutes of Health, Bethesda, MD). The average of two measures showing <1% difference between values was multiplied by slice thickness to obtain muscle volume.

### Muscle biopsy sampling

Muscle samples were obtained under local anesthesia (Carbocain without epinephrine) using the percutaneous biopsy needle technique (Bergström et al., 1967). Samples (~200–300 mg) were taken at rest after an overnight fast and with no previous exercise during the past 48 h from the middle portion of the *vastus lateralis* muscle from the right leg before training (PRE), and from each leg 48 h after the last after training session (POST). Repeated samples were taken in the distal to proximal direction at least 2 cm apart and at the same depth.

### Muscle fiber preparation

A portion of the muscle biopsy (about 5 mg wet weight) was immediately transferred into ice-cold relaxing medium (BIOPS) containing 10 mmol/l Ca^2+^/EGTA buffer, 20 mmol/l imidazole, 50 mmol/l K^+^-4-morpholinoethanesulfonic acid (Mes), 0.5 mmol/l dithiothreitol, 6.56 mmol/l MgCl2, 5.77 mmol/l ATP; 15 mmol/l phosphocreatine at pH 7.1. One to two milligrams of the initial wet weight sample was mechanically dissected using forceps and needles on a small petri dish on an ice-cold glass plate always immerged into BIOPS solution. Next, fibers were incubated in saponin solution (20 μl of 5 mg/ml saponin dissolved into 1 ml BIOPS solution) and gently agitated on a platform shaker for 30 min. Thereafter, fibers were washed for 10 min in ice-cold mitochondrial respiration medium (MiR05; 0.5 mM EGTA, 3 mM MgCl_2_, 60 mM K-lactobionate, 20 mM taurine, 10 mM KH_2_PO_4_, 20 mM HEPES, 110 mM sucrose, and 1 g/l BSA essentially fatty acid free, adjusted to pH 7.1). Fibers were then placed on a paper filter for ~5 s to absorb the extra moisture, before weighed on a microbalance, and transferred into a respirometer chamber.

### Mitochondrial respiration

Mitochondrial respiration was assessed in a high-resolution respirometer (Oroboros Oxygraph, Paar, Graz, Austria). The chambers were sealed using stoppers having rubber rings with the aim to reduce oxygen back-diffusion into the chamber (Steinlechner-Maran et al., 1996). All experiments were performed at 37°C and the polyvinylidene difluoride magnetic stirrers, set at 750 rpm, mixed the chamber content during the experiment. Mitochondrial respiration expressed relative to initial permeabilized fibers wet weight (referred as mitochondrial function) and expressed relative to citrate synthase activity (referred as intrinsic mitochondrial respiration) was obtained by titrating the following substrates into the chambers (final concentrations): octanoylcarnitine (0.2 mM), malate (2 mM) for assessment of leak respiration (EFT_L_), ADP (2.5 mM) to support electron entry from fatty acid β-oxidation through electron-transferring flavoprotein and complex I (EFT_P_), followed by pyruvate (5 mM) and glutamate (10 mM) to stimulate complex I (CI_P_), succinate (10 mM) to stimulate complex I and II linked respiration (CI+II_P_). Carbonyl cyanide m-chloro phenyl hydrazine (0.05 μM steps) was used to measure maximal uncoupled oxidative phosphorylation (Unc). The integrity of the outer mitochondrial membrane was tested by titration of cytochrome c (10 μM).

### Citrate synthase activity assay

Muscle samples were homogenized using a bullet blender in a buffer (39 μl mg^−1^ wet weight) with the following composition (in mM): 50 K_2_HPO_4_, 1 EDTA and 0.05% Triton X-100 adjusted to pH 7.4. The homogenate was centrifuged at 10,000 rpm for 10 min and the supernatant was collected. CS activity was measured in a reagent solution (in mM): 50 Tris-HCl, 0.2 DTNB and 0.1 acetyl-CoA. The reaction was initiated by adding oxaloacetate (7 mM) and the change in absorbance at 412 nm was measured spectrophotometrically (Beckman DU 640) at 25°C.

### Protein extraction and immunoblot analysis

Protein extraction and immunoblot analysis was performed as previously described (Lilja et al., 2017). Briefly, muscle samples were first freeze-dried, cleansed from visible blood, fat and connective tissue and subsequently homogenized in ice-cold buffer (100 μl/mg dry weight) consisting of 2 mM HEPES (pH 7.4), 1 mM EDTA, 5 mM EGTA, 10 mM MgCl_2_, 1% Triton X-100, 2 mM dithiothreitol, and 1.5 % phosphatase and protease inhibitor cocktail (Halt™, Thermo Scientific, Rockford, MD) using a BulletBlender (NextAdvance, Averill Park, NY) with 0.5 mm ZrO beads. The homogenates obtained were subsequently centrifuged at 10,000 g for 10 min at 4°C and the resulting supernatant was collected and stored at −80°C. Protein concentrations were determined in aliquots of supernatant diluted 1:10 in distilled water using the Pierce 660 nm protein assay (Thermo Scientific). Muscle homogenates were diluted with 4 × Laemmli sample buffer (Bio-Rad, Richmond, CA) and homogenizing buffer to obtain a final protein concentration of 1.4 μg/μl. Subsequently, all samples were heated at 95°C for 5 min to denature proteins, and then stored at −20°C until further analysis.

Samples containing total protein of 40 μg were separated using SDS polyacrylamide gel electrophoresis (PAGE) on 26-well Criterion TGX gradient gels (4–20% acrylamide; Bio-Rad). Samples from all three groups were loaded on the same gel. The blots were quantified using Quantity One software version 4.6.3. (BIORAD). To control for appropriate loading and transfer, target proteins were expressed relative to total protein at ~95 KDa obtained by staining the membranes with MemCode Reversible Protein Stain Kit (Thermo Scientific; Antharavally et al., 2004).

The monoclonal primary antibodies used for the detection of target total proteins were the following: total OXPHOS Human Cocktail (no. ab110411; 1:1,000; abcam Cambridge, UK); Anti-Citrate synthetase (CS) (no. ab129095; 1:5,000; abcam Cambridge, UK), Aconitase (ACO2) (no. H00000050-D01P; 1:1,000; Abnova Taipei City, Taiwan), Beclin-1 (no. 3495; 1:1,000; Cell Signaling Technology, Danvers, US), LC3B (no. 3868; 1:1,000; Cell Signaling Technology, Danvers, US), total-ULK1 (no. ab128859; 1:1,000; abcam Cambridge, UK), Phospho-ULK1 (Ser317) (no. 12753; 1:1,000; Cell Signaling Technology, Danvers, US) and LONP1 (no. H00009361-K; 1:1,000; Abnova Taipei City, Taiwan). The secondary antibodies used were all from Cell Signaling Technology: anti-rabbit (1:10,000; total-ULK1, Phospho-ULK1, Beclin-1, LC3B, ACO2, LONP1); and anti-mouse IgG antibodies conjugated with horseradish peroxidase (1:10,000; total OXPHOS Human WB Antibody Cocktail).

### Statistics

Results are presented as mean ± SD. The data were initially assessed for normal distribution and equal variance using Shapiro–Wilk test of normality and Q-Q Plot. Three-way repeated measure analysis of variance (ANOVA) was used to assess differences in dependent variables from baseline measurements, as well as possible leg, group and time interactions. Simple effect tests were employed to follow up significant interactions. A two-tailed *p* < 0.05 was considered significant. To get a better appreciation of the magnitude and precision of the main effects, standardized effect sizes (mean difference/pooled SD) and associated 90% confidence intervals were calculated on the change scores (PRE to POST) and on the difference in change scores across groups (IBU vs. ASA). Training adherence was calculated dividing the attended sessions by the total intended training sessions and expressed in percent. The relationship between the change in CI+II_P_ and the change in muscle volume was assessed using Pearson's product moment correlation analysis. The statistical analyses were carried out using SPSS statistical software version 21 (SPSS Inc., Chicago, Illinois, USA).

## Results

Subject characteristics (i.e., years, body mass, and height) were not significantly different between groups at baseline. Drug compliance was 96 and 97%, whereas the training adherence was 99 and 98%, in the IBU and ASA group, respectively.

### Mass-specific mitochondrial respiration

**Figure 1:**
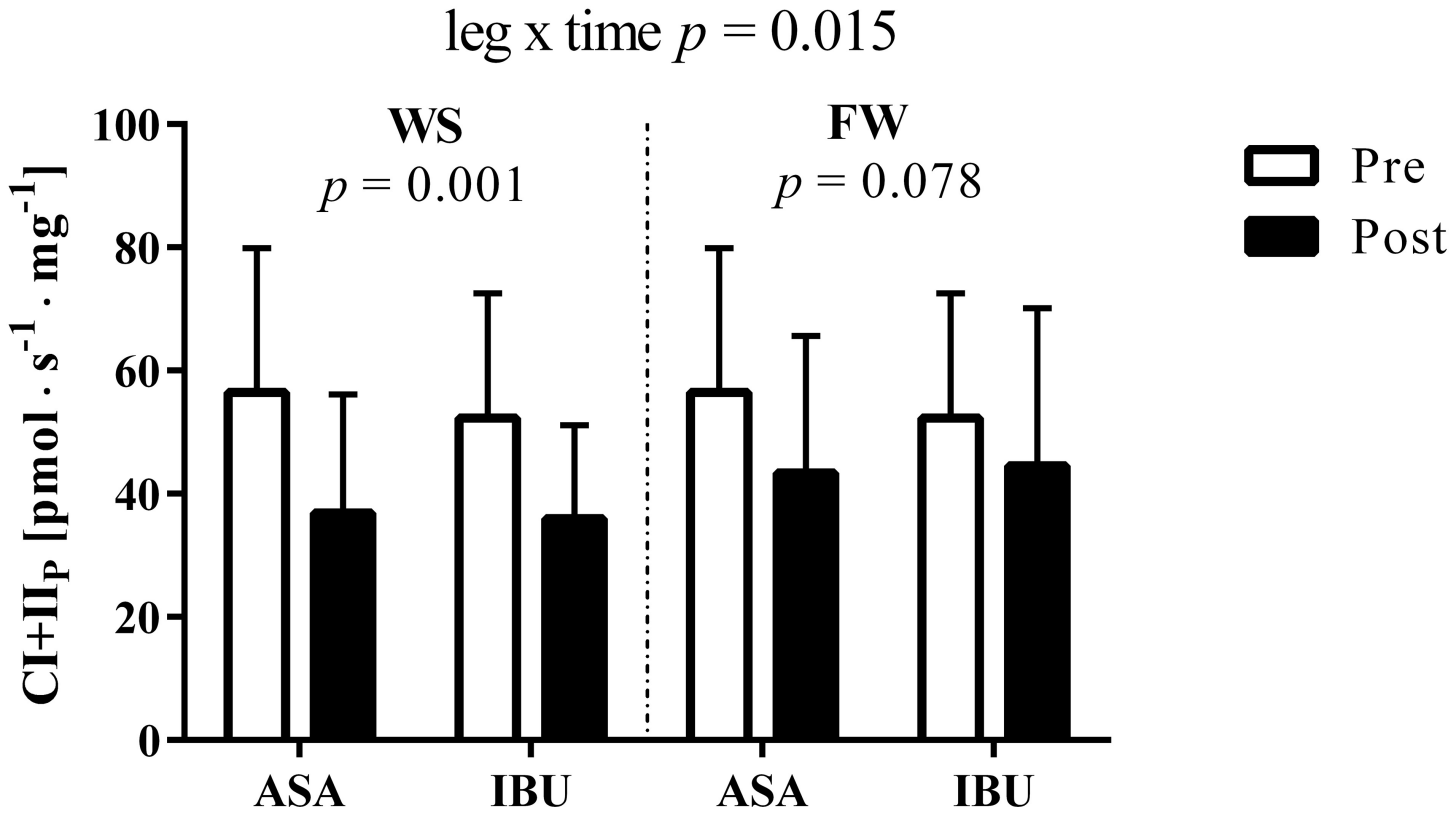
Complex I+II-linked substrate state (CI+II_P_) O_2_ flux [pmol · s^−1^ · mg^−1^] pre and post 8 weeks of resistance training using weight stack (WS) or flywheel (FW) exercise in the acetylsalicylic acid (ASA) and ibuprofen group (IBU).

Although CI+II_P_ decreased in both legs, the decrease was greater (leg × time interaction *p* = 0.015) in WS (33%, *p* = 0.001) than in FW (19%, *p* = 0.078) with no difference across medical treatments (Figure 1). Further, no interactions, no drug effect, and no leg effects were found when analyzing mitochondrial respiration parameters (ETF_L_, ETF_P_, CI_P_, and Unc; Table 1). However, main effects of time were found for ETF_P_ and CI_P_ (*p* < 0.05).

**Table 1:** Mass-specific mitochondrial respiration pre and post 8 weeks of resistance training using weight stack (WS) or flywheel (FW) exercise in the acetylsalicylic acid (ASA) and ibuprofen group (IBU).

	**ASA**			**IBU**		
	**Pre**	**WS post**	**FW post**	**Pre**	**WS post**	**FW post**
ETF_L_ (OctM)	9.85 ± 8.74	4.37 ± 2.96	5.51 ± 5.36	7.44 ± 4.91	4.64 ± 2.53	5.72 ± 3.67
ETF_P_ (OctM)^*^	25.77 ± 12.95	13.50 ± 7.74	15.78 ± 10.74	23.31 ± 16.01	15.14 ± 6.90	17.10 ± 11.65
CI_P_ (OctMPG)^*^	36.44 ± 13.84	25.11 ± 10.08	26.60 ± 14.92	33.93 ± 14.73	23.82 ± 8.56	26.88 ± 14.17
CI+II_P_ (OctMPGS)^*^^#^	56.43 ± 23.47	36.90 ± 19.25	43.38 ± 22.20	52.26 ± 20.29	35.96 ± 15.17	44.57 ± 14.55
Unc (OctMPGSC)	109.44 ± 37.77	75.72 ± 43.35	78.73 ± 76.10	86.56 ± 37.31	62.56 ± 35.68	73.87 ± 57.15

*All values are expressed as means ± SD. O_2_ flux is expressed in (pmol · s^−1^ · mg^−1^ wet weight). Electron-transferring flavoprotein complex (ETF), complex I (CI), complex I+II-linked substrate state (CI+II), and uncoupling state (Unc). LEAK respiration and oxidative phosphorylation are indicated by subscripts L and P, respectively. Substrates are octanoylcarnitine malate (OctM), glutamate (G), pyruvate (P), succinate (S), and Carbonyl cyanide m-chloro phenyl hydrazone (C)*.

* *Main effect of time (P < 0.05)*,

# *leg × time interaction (P < 0.05)*.

### Intrinsic mitochondrial respiration

When mitochondrial respiration was expressed relatively to CS activity (Table 2), ETF_P_ decreased with resistance training (main effect of time *p* = 0.043), and this decrease tended to be significant also for CI_P_ (*p* = 0.082) and CI+II_P_ (*p* = 0.096). CI_P_ also showed a leg x time interaction (*p* = 0.043) since the decrease was mainly evident in the FW leg.

**Table 2:** Intrinsic mitochondrial respiration pre and post 8 weeks of resistance training using weight stack (WS) or flywheel (FW) exercise in the acetylsalicylic acid (ASA) and ibuprofen group (IBU).

	**ASA**			**IBU**		
	**Pre**	**WS post**	**FW post**	**Pre**	**WS post**	**FW post**
ETF_L_ (OctM)	0.21 ± 0.18	0.20 ± 0.23	0.11 ± 0.89	0.28 ± 0.21	0.19 ± 0.12	0.17 ± 0.13
ETF_P_ (OctM)^*^	0.63 ± 0.33	0.52 ± 0.53	0.34 ± 0.16	0.90 ± 0.59	0.58 ± 0.27	0.46 ± 0.33
CI_P_ (OctMPG)^†^^#^	0.94 ± 0.56	0.99 ± 0.82	0.58 ± 0.20	1.28 ± 0.63	0.91 ± 0.39	0.70 ± 0.38
CI+II_P_ (OctMPGS)	1.43 ± 0.88	1.57 ± 1.86	0.96 ± 0.30	1.95 ± 0.84	1.32 ± 0.49	1.09 ± 0.53
Unc (OctMPGSC)	2.87 ± 1.72	3.31 ± 3.97	1.92 ± 0.77	3.45 ± 1.37	2.34 ± 0.98	1.75 ± 1.25

*All values are expressed as means ± SD. O_2_ flux is expressed in (pmol · s^−1^ · CS activity^−1^). Electron-transferring flavoprotein complex (ETF), complex I (CI), complex I+II-linked substrate state (CI+II), and uncoupling state (Unc). LEAK respiration and OXPHOS are indicated by subscripts L, P. Substrate are octanoylcarnitine malate (OctM), glutamate (G), pyruvate (P), succinate (S), and Carbonyl cyanide m-chloro phenyl hydrazone (C)*.

* *Main effect of time (P < 0.05)*,

† main effect of leg (P < 0.05);

# *leg × time interaction (P < 0.05)*.

### CS activity

**Figure 2:**
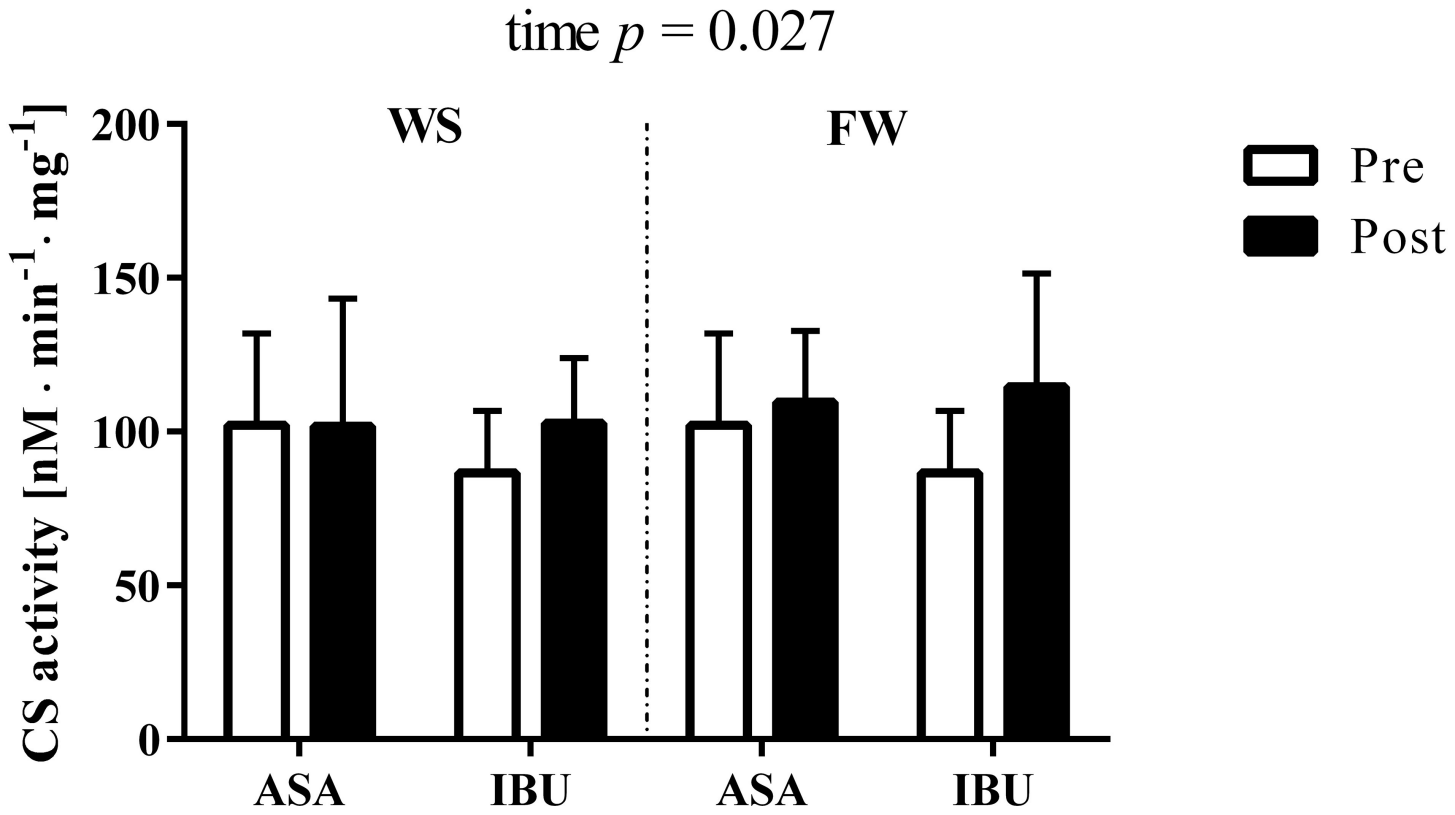
Citrate synthase activity (nM · min^−1^ · mg^−1^) pre and post 8 weeks of resistance training using weight stack (WS) or flywheel (FW) exercise in the acetylsalicylic acid (ASA) and ibuprofen group (IBU).

CS activity increased (main effect of time, *p* = 0.027) with resistance training, with no interactions with medical treatment or training modality (Figure 2).

### Protein expression

**Figure 3:**
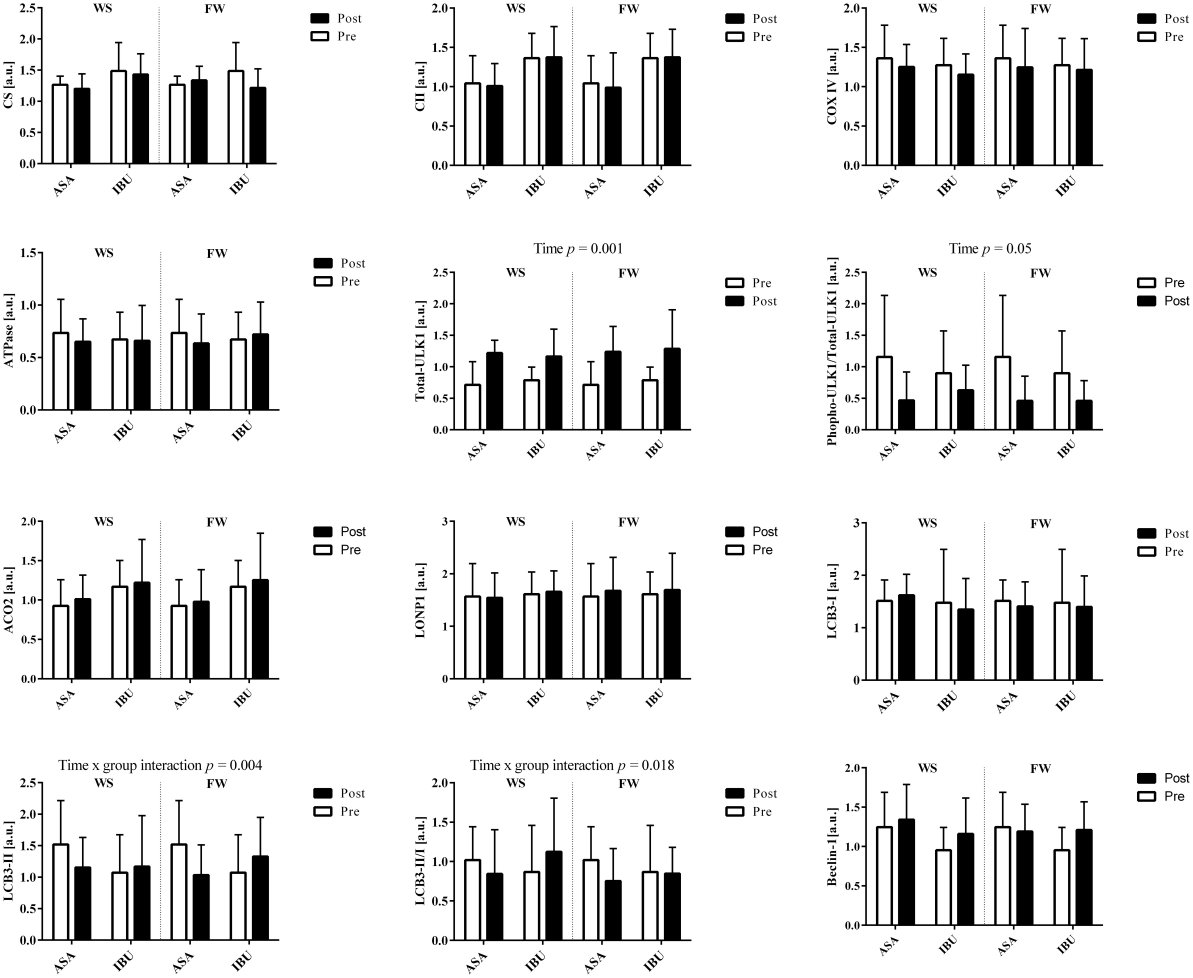
Total protein levels in response to 8 weeks of resistance training using weight stack (WS) or flywheel (FW) exercise in the acetylsalicylic acid (ASA) and ibuprofen group (IBU).

**Figure 4:**
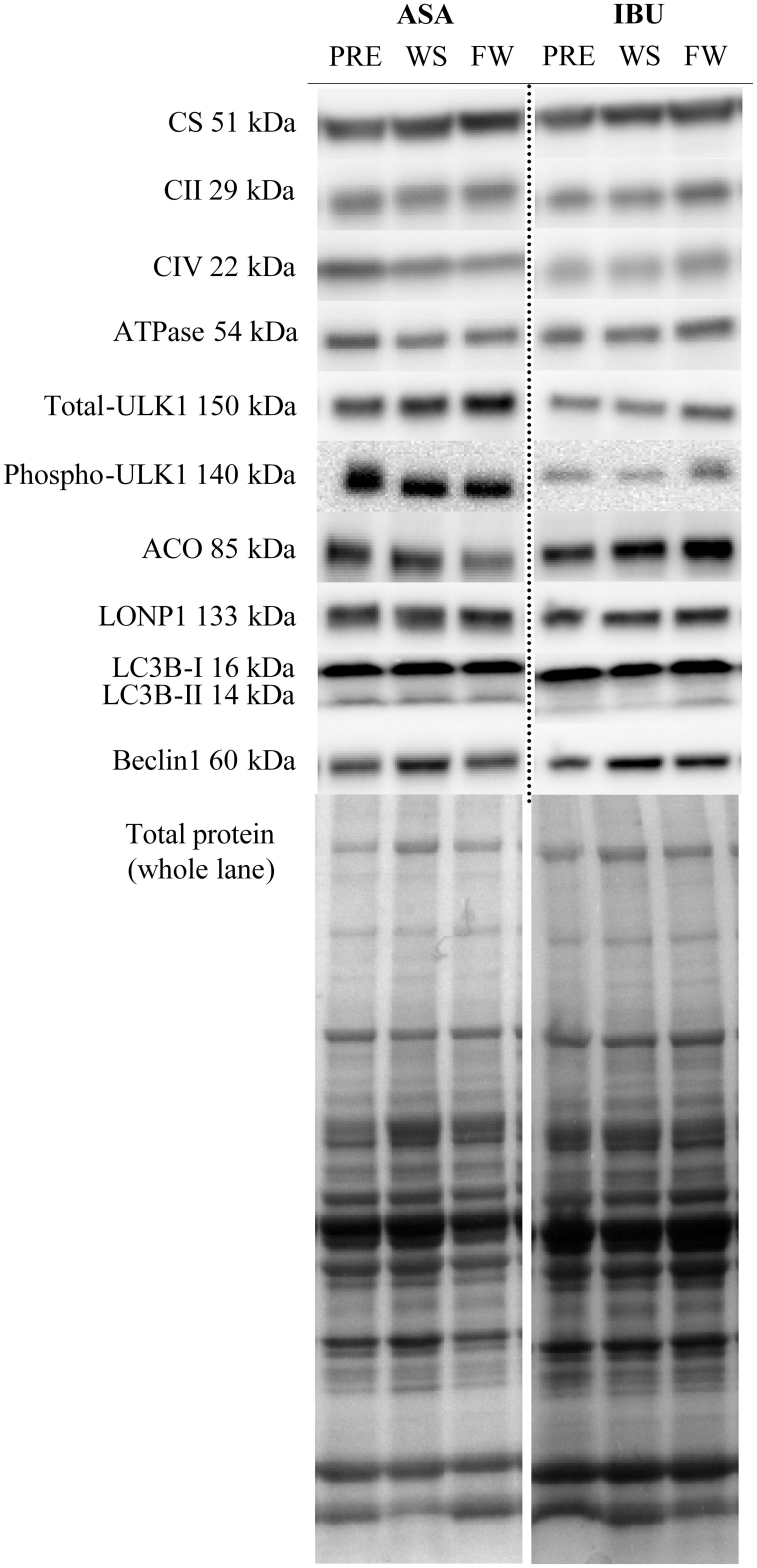
Representative blots of targeted proteins.

There were no group × time or leg interactions for the total protein levels of OXPHOS Human Cocktail (i.e., complex II, IV, V), CS, ACO2, Beclin-1, LC3B-I, LC3B-II, total-ULK1, phospho-ULK1, and LONP1, with the exception for time x group interactions (*p* < 0.05) for LCB3-II and the ratio between LCB3-II/LCB3-I due to a decrease with time in ASA and increased/maintained levels in IBU (Figure 3). Further, there was a time effect for total-ULK1 (~60% increase in both groups; *p* < 0.001) together with a reduction in the phospho-ULK1 /total-ULK1 ratio post training (*p* = 0.005). Also, there was a trend toward a significant increase (*p* = 0.069) in Beclin-1 with training. Representative blots are displayed in Figure 4.

### Correlation between CI+II_P_ and muscle volume

**Figure 5:**
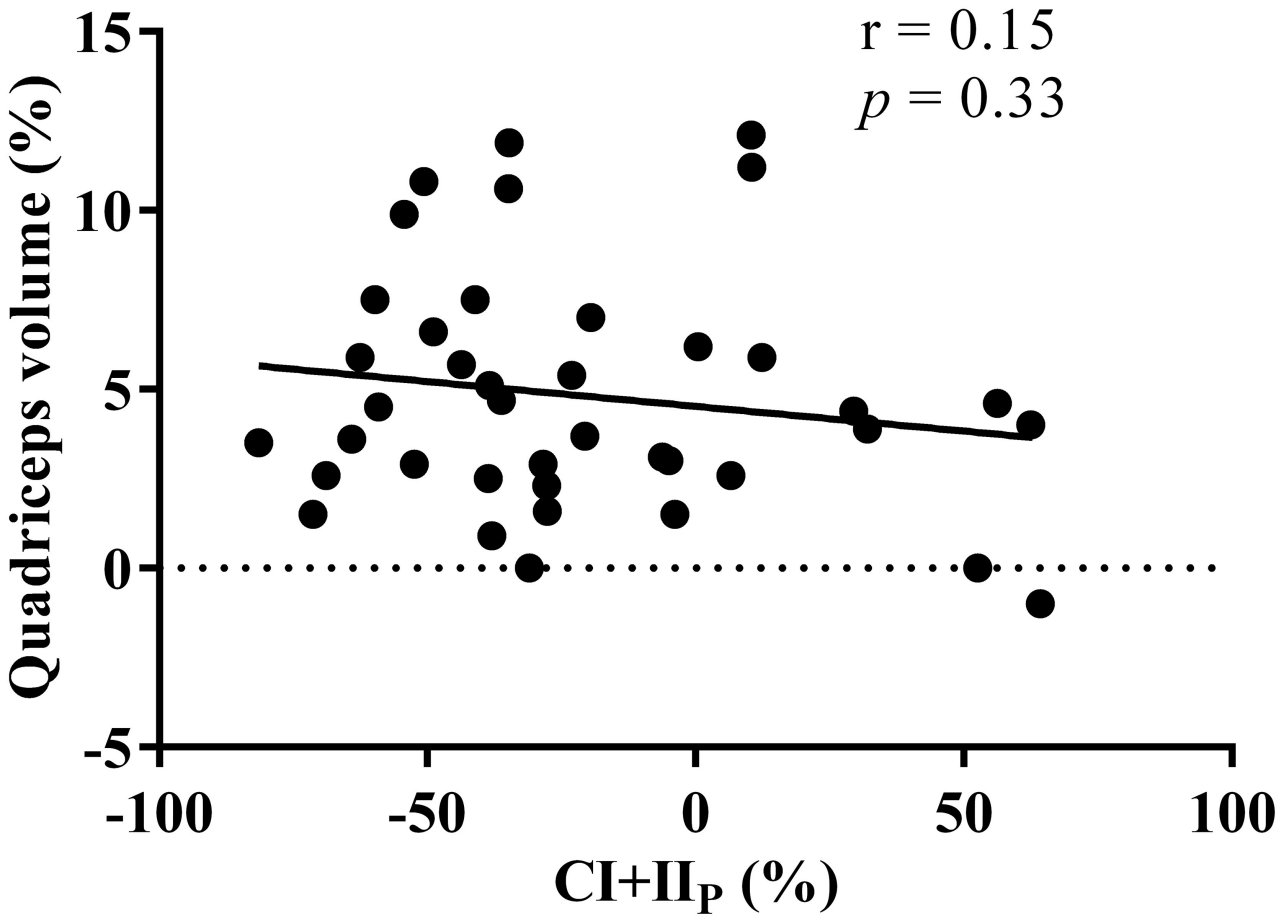
Relationship between the percentage change in maximal oxidative phosphorylation (CI+II_P_) and thigh muscle volume in response to the 8 week resistance training period. *r* indicates the Pearson's product moment correlation factor and *p* the significance levels.

The average increase in muscle volume was 4.8%, yet there was no correlation (*R* = −0.16, *p* = 0.33) between the percentage change in CI+II_P_ and quadriceps muscle volume in response to the 8-week resistance training period (Figure 5).

### Effect sizes

The calculated effect sizes and associated confidence intervals for all mitochondrial parameters are displayed in Supplementary Figure 1.

## Discussion

This is the first study to examine the effects of resistance training, with parallel consumption of high or low daily doses of NSAIDs, on both mitochondrial function and content. Contrary to what was hypothesized, by measuring fully ADP saturated mitochondrial respiration in high resolution respirometry in permeabilized human muscle fibers, we showed that 8 weeks of resistance training, regardless of different doses of NSAID treatment, resulted in reduced mitochondrial function in parallel with increased mitochondrial content in young individuals.

Recently, it was reported that mitochondrial respiration in isolated mitochondria was unaltered (Robinson et al., 2017) by resistance training when reported normalized to initial wet weight and mitochondrial protein levels. However, in the only comparable study available to date which assessed mitochondrial function with the same technique as in the current study, enhanced mitochondrial respiration was reported after 12 weeks of resistance training (Porter et al., 2015). In that study, young healthy participants performed several upper and lower body resistance exercises with 4 exercises targeting legs muscles (3–4 sets of 8–10 repetitions for each exercise at 60–80% 1 RM) 3 times per week for about 70–90 min per session. Thus, although the most obvious difference between studies is the NSAID treatment, it is also possible that the greater training volume along with the 4 weeks longer training intervention could explain the divergent results between Porter et al. and the current findings.

CS activity, a valid marker of mitochondrial content (Larsen et al., 2012), was significantly increased after the resistance training period. Similarly, increased mitochondrial biogenesis was reported in patients with chronic kidney disease following 12-weeks of resistance training (Balakrishnan et al., 2010), and in healthy women after 18 weeks of training (Wang et al., 1993). Yet, reports in young healthy men showed no evidence of increased mitochondrial content (Alvehus et al., 2014; Porter et al., 2015). Thus, it could be that the divergent results are explained by differences in training status, where the metabolic stress with resistance training is higher in individuals with lower fitness, and therefore sufficient to stimulate mitochondrial adaptations to a similar degree as aerobic exercise (Pesta et al., 2011). It is possible that the increased CS activity in the current study may have offset the decrease in mitochondrial respiration since the decreased intrinsic mitochondrial respiration (i.e., mitochondrial respiration normalized to CS activity) was statistically less evident.

Surprisingly in our study, mitochondrial content measured as increased CS activity was not paralleled by changes in total protein levels of mitochondrial electron transfer pathway complexes. Thus, it seems that changes in mitochondrial function following resistance training are not always accompanied by concurrent alterations in markers of skeletal muscle mitochondrial content (Bengtsson et al., 2001; Porter et al., 2015). Additionally, since changes in CI+II_P_ were not correlated with changes in quadriceps volume, our data do not support the hypertrophy-induced mitochondrial dilution theory (MacDougall et al., 1979).

A bulk of scientific evidence show that regular exercise training improves mitochondrial function and content and exerts numerous health benefits (Romanello and Sandri, 2015; Drake et al., 2016). Yet, exercise-induced mitochondrial dysfunction following strenuous exercise regimens was recently reported (Ostojic, 2016). This exercise-induced mitochondrial dysfunction could be caused by aconitase inactivation in the citric acid cycle, as this has been shown to occur following high-volume high-intensity sprint training (Larsen et al., 2015). However, we could not detect any change in total aconitase protein levels. Instead, we found a central autophagy marker, total-ULK1 (Call et al., 2017), significantly upregulated after training, yet a lower level of phospho-ULK1 Ser317 in relation to the total-ULK1. It is under debate whether repeated post exercise mitophagy activation is necessary to induce long term training adaptations (Møller et al., 2015; Schwalm et al., 2017). Although it has been shown that both resistance and endurance training increase mitophagy of peripheral blood mononuclear cells in elderly people (Mejías-Peña et al., 2016, 2017), mitophagy in muscle tissue may act through a different mechanism. In our study, to assess if mitophagy signaling was upregulated with training, different proteins involved in the lysosome formation were measured (i.e., Beclin-1, LCB3-I, and LCB3-II; Klionsky et al., 2016). No changes in these proteins were found except for a trend toward a significant upregulation of Beclin-1, indicating that total-ULK1 mediated autophagy may have had downstream effects on Beclin-1 in the attempt to regulate mitochondrial turnover. However, since mitochondrial function was reduced with training in parallel with increased mitochondrial content, we speculate that dysfunctional mitochondria were not degraded in this cohort of young subjects. Rather, impaired mitochondria may have accumulated (Navratil et al., 2008) and thus caused the increased mitochondrial content.

The rate of mitochondrial ROS emission has been shown to be inversely proportional to the rate of respiration. Thus, increased respiration results in reduced ROS production (Picard et al., 2011). Therefore, it seems unlikely that excessive ROS production due to high O_2_ flux rate was the cause of exercise-induced mitochondrial dysfunction in the current study. Instead, it seems more likely that the impaired intrinsic mitochondrial function was caused by inflammatory responses induced by the resistance exercise bouts, e.g., muscle damage with excessive ROS production (despite NSAID treatment; Handa et al., 2014) due to neutrophil activation and macrophage infiltration (Kozakowska et al., 2015). However, this is only a speculation since no ROS markers were assessed in this study. Yet, our data do not support mild mitochondria uncoupling as a plausible strategy employed by the mitochondria to reduce the ROS production and balance mitochondrial physiology (Schrauwen and Hesselink, 2004; Hesselink et al., 2016) since mitochondria uncoupled when exposed to low titrations of protonophore (i.e., Carbonyl cyanide m-chloro phenyl hydrazine). Nevertheless, it remains a possibility that the decreased mitochondrial respiration only reflects a temporary phase specific to the regulatory events occurring at the selected biopsy time point (i.e., 48 h after the last exercise bout). Thus, future studies should also include a later biopsy to ensure true basal conditions.

In contrast to the hypothesis, high doses of NSAID intake did not negatively affect mitochondrial function compared with low doses. This notion is in contrast to the ROS theory causing exercise-induced mitochondrial dysfunction since NSAIDs have been reported to increase mitochondria-derived ROS and cause cell damage in humans (Handa et al., 2014). Our results do indicate, however, that flywheel resistance training, emphasizing eccentric overload, rescued some of the reduction in mitochondrial function seen with conventional resistance training. It is difficult to delineate the specific mechanism for this effect since FW and WS training hold several differences. It could be speculated, however, that the greater cytokine and leukocyte response following a single exercise bout with high eccentric loads (i.e., flywheel exercise) could, after repeated training, have led to a reduced overall stress and ROS production following FW compared to WS training (Paulsen et al., 2012).

This study was not without limitations. The inclusion of a non-treated control group and a drug-only group would have given us greater confidence in contrasting the effects of the training vs. the NSAIDs. With the current design, we cannot completely rule out an accumulating effect of ASA treatment in skeletal muscle over time. It was also unfortunate that we could not obtain fresh muscle tissue samples from all of the subjects in the original study. Consequently, the rather low sample size obviously increases the risk of type 2 errors and random effects. We also appreciate that the high-resolution respirometry has its limitations and that analysis in isolated mitochondria, and/or analysis in a more stressful condition, could have yielded different results (Irving et al., 2015; Groennebaek and Vissing, 2017). Likewise, since ibuprofen is a non-selective COX-inhibitor, it remains a possibility that using selective COX-inhibitors instead could have revealed more information about the possible regulatory mechanisms. Altogether, it is clear that more research is needed in the area before more finite conclusions can be drawn regarding the potential of resistance training and/or NSAIDs to stimulate or interfere with mitochondrial adaptations.

In summary, we show that 8 weeks of resistance training with both high and low doses of NSAID intake reduced mitochondrial function but not mitochondrial content in healthy young individuals. Interestingly, the decreased mitochondrial function with resistance exercise was not exacerbated by high doses of NSAID consumption, suggesting that the resistance training was the primary mediator of this effect rather than the NSAIDs. Finally, we show that flywheel resistance training, emphasizing eccentric overload, could counteract some of the reduction in mitochondrial function seen with conventional resistance training.

## Author contributions

DC, ML, MM, FL, TG, and TL: all contributed to the conception and design of the experiment; DC, ML, and MM: collected data; DC and TL: analyzed data; DC: prepared the figures and tables; DC and TL: drafted the manuscript, which was revised critically by all co-authors; All authors read and approved the final version of the manuscript. All persons designated as authors qualify for authorship, and all those who qualify for authorship are listed.

### Conflict of interest statement

The authors declare that the research was conducted in the absence of any commercial or financial relationships that could be construed as a potential conflict of interest.
