# Supplementary figures and images for: Resistance Training with Co-ingestion of Anti-inflammatory Drugs Attenuates Mitochondrial Function
Source: Front Physiol. 2017 Dec 19;8:1074. doi: 10.3389/fphys.2017.01074 (PMC5742251; doi:10.3389/fphys.2017.01074)

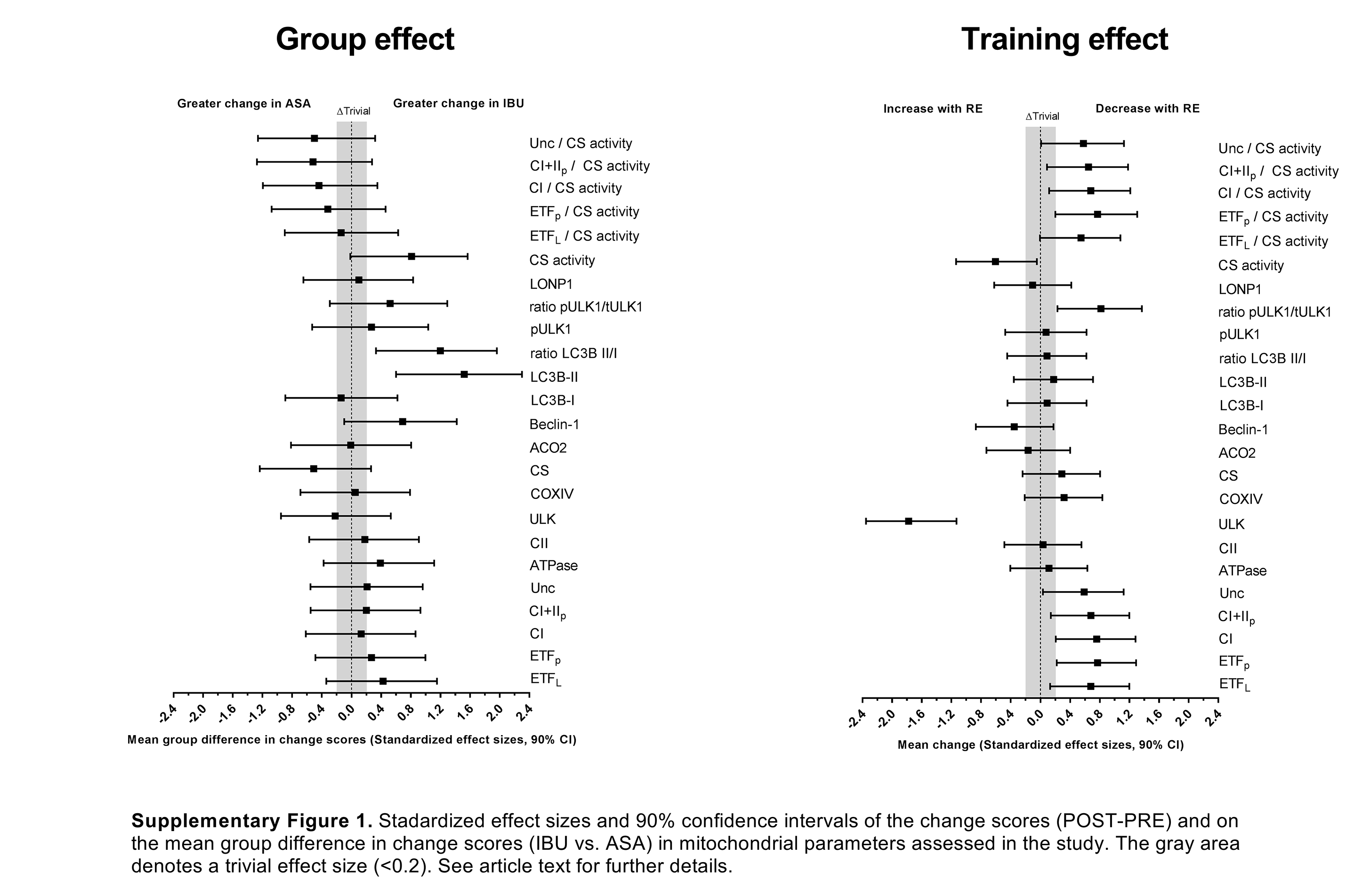

Supplement: Supplementary file 1 [file Image1.TIF]
